# Individual Dispositions and Situational Stressors in Competitive Sport: The Role of Stress Mindset in the Cognitive Appraisals Processes

**DOI:** 10.3389/fpsyg.2022.829053

**Published:** 2022-05-24

**Authors:** Dajana Čopec, Matea Karlović Vragolov, Vesna Buško

**Affiliations:** ^1^Mental Training Center d.o.o., Zagreb, Croatia; ^2^Department of Kinesiological Anthropology and Methodology, Faculty of Kinesiology, University of Zagreb, Zagreb, Croatia; ^3^Department of Psychology, Faculty of Humanities and Social Sciences, University of Zagreb, Zagreb, Croatia

**Keywords:** stress, cognitive appraisals, personality dimensions, stress mindset, competitive sport

## Abstract

Personality has widely been documented to play an important role in the cognitive appraisal and stress processes. Emerging studies highlight the stress mindset as a new concept that could add to the understanding of individual differences in stress experiences. This study aimed to examine the relative contribution of Big Five personality dimensions and stress mindset in accounting for measures of cognitive appraisals of stress among the competing athletes. The study was conducted on a sample of 125 collegiate athletes of both genders who actively compete in sport. All the participants were regular undergraduate or graduate students at the Faculty of Kinesiology of the University of Zagreb. A questionnaire including demographic information about athletes and their sport career, stress mindset measure (SMM), situation-specific cognitive appraisal scale, sources of stress scale, and personality scale measured by IPIP-50 was administered in an online form using the Google Forms platform. Multivariate hierarchical regression procedures resulted in somewhat different predictor structures accounting for cognitive appraisals of threat, loss, and challenge, used as criterion variables. The set of Big Five personality dimensions and stress mindset measure proved to have a significant additive contribution to the explanation of each of the three cognitive appraisal criterion variances. The study results support the current body of literature suggesting a unique role of the stress mindset construct in explaining individual differences in cognitive stress appraisal among athletes above and beyond general personality dimensions.

## Introduction

It is widely understood that experiencing stress can have both positive and negative effects on wellbeing in various aspects of one's life (Folkman and Moskowitz, 2000), sport being just one of them. It appears that due to the challenging and result-orientated nature of sport (McCarthy and Giges, 2017), stress is almost an inevitable experience among athletes, especially those that regularly compete (Scanlan et al., 1991). The effects of stress on athletes are, however, diverse. Stress can aid to athlete's determination and motivation (Fletcher et al., 2011), but it can also lead to burnout (Gustafsson et al., 2011), overtraining (Meehan et al., 2004), and poor performance (Gould et al., 1999). Therefore, it is not surprising that appropriate ways of managing stress are necessary for experiencing success in sport (Hoar et al., 2006).

According to the dominant conceptual framework for the analysis of stress processes—transactional theory of stress and coping (Lazarus and Folkman, 1984, 1987; Lazarus, 2000, 2006), stress includes a transaction between the person and the situation that one finds challenging, as well as the judgment of personal resources for handling these challenges (Lazarus and Folkman, 1984, 1987). The theory introduced the concepts of cognitive appraisals as central processes connected to experiencing stress. Cognitive appraisal stands for the evaluation of the meaning the environmental factors have on one's wellbeing. There are two main types of appraisals, namely, primary and secondary appraisals. Primary appraisal relates to the assessment of the complexity of the situation and the meaning it has on one's wellbeing (Folkman, 1984, 2010). According to Lazarus and Folkman (1984), there are three types of primary appraisals—the situation can be assessed as irrelevant for an individual, benign-positive with a potential to enhance wellbeing, or stressful, that is, the situation presents a threat to wellbeing. The situation appraised as stressful can be related to the perception of harm/loss (the situation has already left damage to one's wellbeing), threat (the damage is possible), or challenge (the situation can lead to growth and development). The secondary appraisal is related to the evaluation of one's resources for dealing with the situation and the level of control over it that one possesses (Folkman, 1984).

It is worth pointing out the subjective and situational nature of cognitive appraisals. An objectively same situation can be appraised differently depending on one's resources and various psychological and social characteristics (Lazarus, 2006). Several personal (e.g., self-confidence and personality) and situational (e.g., the predictability and novelty of the situation) characteristics have typically been reported as important antecedents to cognitive appraisals. Research has found significant relationships between personality and cognitive appraisals. For instance, higher neuroticism was found to be associated with experiencing stress of higher intensity, lower perception of control over the stressors, and a higher chance of appraising stressful situations as threatening (Shewchuk et al., 1999; DeLongis and Holtzman, 2005; Kulenović and Buško, 2006; Semmer, 2006; Tong et al., 2006). On the contrary, conscientiousness, agreeableness, openness, and in part extraversion were reported to be associated with appraising situations as challenging, as well as with higher perception of control and available resources (Penley and Tomaka, 2002; Semmer, 2006; Schneider et al., 2012).

Stress mindset appeared in recent literature as a construct that could advance the understanding of stress experiences. It has been defined as the extent to which one believes that stress has enhancing (“stress-is-enhancing mindset”) or debilitating consequences (“stress-is-debilitating mindset”) in a range of life's areas (e.g., health, productivity; Crum et al., 2013). The authors suggest that our experiences of stressful events depend on whether we believe that stressful experiences are generally threatening or represent an opportunity for progress. Compared with situation-specific account of cognitive appraisal, stress mindset refers to the general view on stress. A person can evaluate an event as a stressful and threatening one (cognitive appraisal) but believe that positive outcomes are possible and the whole experience will make one stronger (“stress-is-enhancing mindset”; Crum et al., 2013, 2017). Thus, a stress-is-enhancing mindset seems to be linked to better cognitive flexibility when dealing with stressors and lower levels of cortisol (Crum et al., 2013). Kilby and Sherman (2016) found that in comparison with the ones holding stress-is-debilitating mindset, the ones holding stress-is-enhancing mindset more often appraise stressful events as a challenge. Recent studies have found that a stress-is-enhancing mindset is connected to experiencing stress less intensely among students (Keech et al., 2018) and police officers (Keech et al., 2020). Stress mindset has also been linked to depression, anxiety, and wellbeing (Jiang et al., 2019; Huebschmann and Sheets, 2020). There seems that a stress-is-enhancing mindset could potentially have a soothing effect, i.e., a positive effect on wellbeing when one is experiencing stress.

This research examines the relative contribution of personality dimensions and stress mindset in explaining stress appraisals in athletes. As mentioned before, sport represents a challenging environment, and athletes are regularly exposed to various organizational (e.g., leadership and quality of the environment in which they train), competitive (e.g., preparation and rivalry), and personal (e.g., balancing sport and private life) sources of stress (Wagstaff et al., 2019). Therefore, gaining insight into the relevant antecedents of stressful experiences in the sports setting seems to be conceptually and practically valuable. It is theoretically interesting and important to evidence whether this new concept of stress mindset plays a unique role in accounting for stress appraisals over and above well-known personality dimensions. Given the suggested nature of the stress mindset construct, eventual findings on its relevance in explaining athletes' stress experiences could then be useful in fostering more efficient stress management strategies.

## Methods

### Participants

The participants were student athletes from the Faculty of Kinesiology, University of Zagreb. All participants were actively involved in sports at the time the research was conducted. The sample consisted of 125 students (*M* = 22.31 years, *SD* = 2.27, 32% female). In terms of the level of sport performance, 58 (46.4%) athletes in the sample participated in sport at the amateur level, 58 (46.4%) at the semi-professional level, and 9 (7.2%) at the professional level. The highest percentage (38.4%) of participants were involved in their sport for 6–10 years. Most participants (45.6%) trained every day of the week. Regarding the level of competition, 46 (36.8%) participants competed at the country level, 47 (37.6%) at the national level, and 18 (14.4%) at the international level, with 14 (11.2%) participants reported as not competing at the time of the data collection. Slightly less than half of the participants stated that they competed once a week in the past 6 months (*N* = 61; 48.8%).

### Measures

#### Stress Mindset Measure

The stress mindset measure (SMM; Crum et al., 2013) was used to assess participants' general way of thinking about stress. Participants were instructed to rate how strongly they agree with eight statements on a 5-point Likert type scale (0 “strongly disagree” to 4 “strongly agree”). The total score was computed as the sum of scores on all items, varying in the theoretical range from 0 to 32. A higher score on the scale indicates more enhancing beliefs about stress and a lower one indicates more debilitating beliefs about stress. For the purpose of this research, the scale was translated into Croatian using the back-translation method. This adaptation replicated previous findings on the dimensionality of the measure and showed high reliability of the scale on the Croatian sample (Cronbach's alpha, α = 0.91).

#### Sources of Stress

To identify the main sources of stress in participants' recent sport experience, we administered a list of potential stressors classified into the following seven categories: injuries, rivalry, poor performance, training for competition, pressure to prove oneself, high self-expectations on performance, and personal desire to prove oneself. Participants were instructed to choose one of those seven briefly described categories of problems appraised as most stressful in the context of their competitive sport participation during the past 6 months. The list was based on a comprehensive analysis of published papers conducted by Wagstaff et al. (2019). Keeping in mind the selected category of sources of stress, the participants approached the further part of the questionnaire.

#### Primary Cognitive Appraisal

Appraisals of event stressfulness were measured by three 6-item scales of emotions reflecting loss, threat, and challenge appraisals (Kulenović and Buško, 2006). Items were rated on a 4-point intensity scale ranging from 0 “not at all” to 3 “very much” with reference to the selected sources of stress in competitive sports. Total scores on each scale were computed as the sum scores on respective items, varying in theoretical range from 0 to 18. Cronbach's alpha internal consistencies obtained in this research were 0.81, 0.77, and 0.82 for the loss, threat, and challenge appraisals, respectively.

#### International Personality Item Pool

Croatian translation of the 50-item International Personality Item Pool was administered (IPIP, Goldberg, 1999; Mlačić and Goldberg, 2007). The measure consists of 50 short statements intended to assess each of the Big Five personality dimensions: extraversion, agreeableness, conscientiousness, emotional stability, and intellect. Each dimension is encompassed by 10 items rated on a 5-point Likert type scale from 1 “completely incorrect” to 5 “completely true.” The Cronbach's alpha coefficients in this study were as follows: α = 0.85 for extraversion, α = 0.84 for agreeableness, α = 0.82 for conscientiousness, α = 0.87 for emotional stability, and α = 0.65 for intellect.

The survey form also included several demographic items: age, gender, and information about some aspects of participants' sport experience [i.e., sport, level of sport performance, years of sports participation, training frequency (per week), level of competition, and frequency of competing in the past 6 months].

### Procedure

The study was conducted in June–July 2020, during the first lockdown period due to COVID-19 pandemic. Google Forms platform was used for data collection. The target population of active athletes attending the Faculty of Kinesiology in Zagreb were recruited by invitation for participation sent *via* e-learning system and students' social networks (Facebook). Participants followed a link to the online survey. Along with basic information on the study purpose and general instructions given on the first page of the instrument, participants were guaranteed anonymity of their contribution and were informed about the option to withdraw from the study at any time. The final sentence of the instruction page informed respondents that by selecting the “Next” button, they confirmed that they had read the information about the research and that they were willing to participate in it. The research project under which the study was conducted has been approved by the Ethics Committee of the Faculty of Humanities and Social Sciences, University of Zagreb.

### Data Analysis

Along with descriptive and reliable statistics of all the study variables, canonical discriminant analysis was performed to examine the extent of differences in selected background, individual dispositions, and cognitive appraisal variables among groups defined by category of stressors declared by participants. Hierarchical regression procedures were implemented in answer to the main study question. All the analyses were done using IBM SPSS statistical software, v. 26.0.

## Results

The study aimed to examine the relative importance of personality dimensions and stress mindset in accounting for three stress appraisal measures in athletes. Main descriptive statistics and intercorrelations of all the study variables are given in Table 1. Relatively low average scores are obtained on all cognitive appraisal measures, whereas the mean stress mindset score is positioned close to the center of the theoretical range of the composite scale, suggesting no inclination toward the positive or negative way of thinking about stress. Average personality scale scores seem to correspond reasonably well to figures usually found in students athlete population. Certain departure from normality was observed for several scales as indicated by shapes of distributions and Kolmogorov-Smirnov *z*-values. However, the indices of skewness and kurtosis varied within a tolerable range (from −0.89 to 0.89 for skewness, and from −0.97 to 0.97 for kurtosis), which made the execution of the planned multivariate analyses justified.

**Table 1:** Main descriptive statistics and intercorrelations of the study variables.

	**1**	**2**	**3**	**4**	**5**	**6**	**7**	**8**	**9**	**10**	**11**	**12**	**13**
(1) Gender	–												
(2) Level of performance	0.10	–											
(3) Training frequency	0.04	0.69^**^	–										
(4) Competition frequency	0.10	−0.10	0.03	–									
(5) Extraversion	0.06	−0.01	0.04	0.03	–								
(6) Agreeableness	−0.39^**^	−0.06	0.03	0.06	0.06	–							
(7) Conscientiousness	−0.35^**^	−0.20^*^	−0.20^*^	−0.06	−0.05	0.27^**^	–						
(8) Emotional stability	−0.03	0.09	0.07	0.14	0.47^**^	0.08	0.16	–					
(9) Intellect	−0.10	−0.18^*^	−0.14	−0.09	0.13	0.11	0.29^**^	0.01	–				
(10) Stress mindset	−0.08	0.08	0.02	0.13	0.37^**^	0.12	−0.00	0.45^**^	−0.15	–			
(11) Cognitive appraisal of loos	0.03	0.04	−0.04	−0.10	−0.16	−0.13	−0.06	−0.47^**^	0.06	−0.43^**^	–		
(12) Cognitive appraisal of threat	−0.20^*^	0.07	0.10	0.01	−0.30^**^	0.13	−0.08	−0.39^**^	−0.08	−0.38^**^	0.58^**^	–	
(13) Cognitive appraisal of challenge	0.05	0.05	−0.06	0.18^*^	0.23^**^	0.06	0.13	0.23^*^	0.06	0.55^**^	−0.16	−0.17	–
M (SD)	–	–	–	–	35.22 (7.40)	40.22 (6.28)	37.78 (6.86)	36.21 (7.46)	38.25 (4.96)	15.34 (8.20)	4.14 (3.69)	6.10 (3.35)	6.14 (4.26)
Theoretical range	1–2	1–3	1–5	1–6	10–50	10–50	10–50	10–50	10–50	0–32	0–18	0–18	0–18
Total range	1–2	1–3	1–5	1–6	13–50	23–50	21–50	18–50	27–48	0–32	0–15	0–16	0–16
Cronbach's α	–	–	–	–	0.85	0.84	0.82	0.87	0.65	0.91	0.81	0.77	0.82
K–S	–	–	–	–	0.06	0.10^**^	0.07	0.07	0.08	0.10^**^	0.17^**^	0.13^**^	0.10^**^

* *p < 0.05*,

** *p < 0.01; gender codes: 1, females; 2, males; Level of performance codes: 1, professional; 2, half-professional; 3, amateur; K–S, Kolmogorov–Smirnov z-statistic*.

Consistent with general postulates of the transactional stress and coping theory, we examined whether the differences can be observed in individual disposition, situation appraisal, and sport-related background variables depending on the sorts of stressors participants declared as most taxing in the given timeframe. Apart from the theoretical rationale behind this question, the statistical treatment of the data concerning the main problem of this research depended on the outcomes of these analyses. Canonical discriminant analysis was performed to test the multivariate differences between 7 groups of participants defined by the selected categories of stressors. The analysis revealed statistically significant albeit not particularly marked intergroup differences with only one significant discriminant function accounting for 37% of total intergroup variability (Wilks' Λ = 0.388, λ = 0.392, r_c_ = 0.531, *p* < 0.05). Standardized discriminant function and structure coefficients suggested that the contribution of cognitive appraisal, disposition, and background measures to intergroup differences were modest (e.g., r's of 0.40 and −0.323 for intellect and challenge appraisals, respectively), or negligible (e.g., r's of −0.069 and 0.175 for stress mindset and threat appraisals, respectively).

As shown in Table 2, certain intergroup separation can be observed, especially for the second group compared with the third and seventh categories of stressors, as indicated by the positions of the group centroids on the derived discriminant function. However, taking into account rather small group sizes and considerable overlap of their score distributions, along with mostly insignificant univariate tests of differences among the groups on the same set of variables, we deemed reasonable to assume that the choice of stressor type did not present relevant source of sample heterogeneity in terms of cognitive appraisals and personality structure examined. Hence, all the succeeding regression analyses were performed on the total sample.

**Table 2:** Positions of group centroids on the first canonical discriminant function.

				**Discriminant function 1**
**Group**				**Group centroid**
	**Selected category of stressors**	***n***	**%**	
1	Injuries	31	24.8	0.040
2	Rivalry	8	6.4	1.525
3	Poor performance	25	20.0	-0.499
4	Training for competition	9	7.2	-0.014
5	Pressure to confirm oneself	16	12.8	0.622
6	High expectations on performance	12	9.6	0.515
7	Personal desire to prove oneself	24	19.2	-0.707

*N = 125; n, group sizes*.

To examine the contribution of personality dimensions and stress mindset in explaining the cognitive appraisal variance, three hierarchical regression analyses were performed. The analyses were performed in three steps. To control for the main background and sport-related variables, the first step included gender, level of sport performance, training frequency, and competition frequency. The choice of the background variables was governed by research findings on the relationships of these variables with the stress experiences in competitive sports (Mellalieu et al., 2006). In the second step, five personality dimensions were added, and the third step included just the stress mindset variable. The results of these analyses are shown in Table 3.

**Table 3:** Hierarchical regression analyses: the additive role of personality dimensions and stress mindset in accounting for cognitive appraisals of stress.

**Sets of predictor variables**	**Step 1**	**Step 2**	**Step 3**
	**β**	**β**	**β**
	**Criterion: cognitive appraisal of challenge**		
Gender	0.01	0.07	0.13
Level of performance	0.21	0.24	0.16
Training frequency	−0.21	−0.21	−0.14
Competition frequency	0.21^*^	0.20^*^	0.16^*^
Extraversion		0.20^*^	0.07
Agreeableness		0.04	−0.01
Conscientiousness		0.15	0.18^*^
Emotional stability		0.07	−0.12
Intellect		0.02	0.12
Stress mindset			0.58^**^
*R^2^*	0.06	0.15^*^	0.38^**^
ΔR^2^	0.06	0.09^*^	0.23^**^
	**Criterion: cognitive appraisal of threat**		
Gender	−0.20^*^	−0.20^*^	−0.23^*^
Level of performance	0.05	0.08	0.12
Training frequency	0.03	0.08	0.10
Competition frequency	0.07	0.05	0.01
Extraversion		−0.14	−0.06
Agreeableness		0.11	0.13
Conscientiousness		−0.09	−0.10
Emotional stability		−0.35^**^	−0.25^**^
Intellect		−0.03	−0.09
Stress mindset			−0.31^**^
*R^2^*	0.05	0.25^**^	0.32^**^
Δ*R*^2^	0.05	0.21^**^	0.07^**^
	**Criterion: cognitive appraisal of loss**		
Gender	0.03	−0.03	−0.06
Level of performance	0.10	0.18	0.22
Training frequency	−0.09	0.02	0.04
Competition frequency	−0.11	−0.11	−0.15
Extraversion		0.10	0.17
Agreeableness		−0.11	−0.09
Conscientiousness		0.05	0.04
Emotional stability		−0.53^**^	−0.43^**^
Intellect		0.06	−0.01
Stress mindset			−0.31^**^
*R^2^*	0.02	0.26^**^	0.32^**^
Δ*R*^2^	0.02	0.24^**^	0.07^**^

* *p < 0.05*,

** *p < 0.01; N = 125*;

Δ*R^2^, change in the explained criterion variance after a new block of variables was entered into equation; β, standardized regression coefficients*.

In the first hierarchical regression analysis, the criterion was the cognitive appraisal of challenge. The set of sport-related and background variables, entered in the first step of the analysis, did not account for a significant portion of criterion variance, albeit the individual contribution of competition frequency reached a significance level (β = 0.21, *p* < 0.05). Introducing personality dimensions in the second step of the analysis managed to increase the explained criterion variance (Δ*R*^2^ = 0.09, *p* < 0.05) with extraversion as the only predictor variable with marginally significant contribution (β = 0.20, *p* = 0.05). The stress mindset variable introduced in the third step explained an additional, statistically and substantially significant amount of variance of the challenge appraisal (Δ*R*^2^ = 0.23, *p* < 0.01). Along with modest contribution of competition frequency (β = 0.16, *p* < 0.05) and conscientiousness (β = 0.18, *p* < 0.05), stress mindset appeared as the strongest predictor of challenge appraisals in the final regression equation (β = 0.58, *p* < 0.01). Thus, athletes who competed more frequently score higher on conscientiousness, and those who think more positively about stress are more likely to perceive stressors as a challenge. The entire model explained approximately 38% of the challenge appraisal variance [*F*(10, 114) = 7.08, *p* < 0.001].

In the second hierarchical regression analysis, cognitive appraisal of threat was included as a criterion. Again, the first step model did not reach a significance level, although regression weight for gender appeared marginally significant (β = 0.20, *p* < 0.05). In the second step of the analysis, the set of personality dimensions explained a significant portion of the additive criterion variance (ΔR^2^ = 0.21, *p* < 0.01). Along with gender (β = 0.20, *p* < 0.05), emotional stability (β = −0.35, *p* < 0.01) proved as a significant predictor in this step. The stress mindset was added to the equation in the final step with the additional 7% of the explained criterion variance (*p* < 0.01). In this step, along with stress mindset (β = −0.31, *p* < 0.01), emotional stability (β = −0.25, p <0.01), and gender (β = −0.23, *p* < 0.05) remained as statistically significant individual predictors, such as in the previous step. Thus, male athletes, those who thought more positively about stress and who score higher on trait emotional stability, were less likely to experience stressors as threatening. The set of predictor in the final regression equation explained 32% of the threat appraisal variance [*F*(10, 114) = 5.37, *p* < 0.01].

In the third hierarchical regression analysis, cognitive appraisal of loss was included as a criterion. Predictors entered in the first step did not account for a significant portion of criterion variance. Personality dimensions introduced in the second step significantly increased the explained criterion variance (Δ*R*^2^ = 0.24, *p* < 0.01), and emotional stability (β = −0.53, *p* < 0.01) was shown to be the only significant predictor. After the stress mindset was entered in the third step of the analysis, an additional 7% of loss appraisal variance was explained (Δ*R*^2^ = 0.07, *p* < 0.01). Emotional stability (β = −0.43, *p* < 0.01) was shown as independent predictor, along with the stress mindset (β = −0.31, *p* < 0.01). Thus, following the figures in the final equation, athletes with lower scores on the emotional stability dimension (β = −0.43, *p* < 0.01), and those who think more negatively about stress (β = −0.31, *p* < 0.01) are more likely to perceive stressors as a loss. The whole model accounted for 32% of the loss appraisal variance [*F*(10, 114) = 5.42, *p* < 0.01].

## Discussion and Conclusion

This research dealt with potential sources, types, and intensity of stress athletes tend to experience in competitive sport. Grounded in the transactional theory of stress (Lazarus and Folkman, 1984, 1987; Lazarus, 2000, 2006), the study aimed to examine the role of personal antecedents of the stress processes in accounting for the stressful experiences in athletes. In addition to personality dimensions as widely established and theoretically relevant antecedents, we sought to examine the additive contribution of a relatively novel construct of stress mindset (Crum et al., 2013, 2017) in explaining individual differences in cognitive appraisals of loss, threat, and challenge in athletes.

Consistent with the basic hypotheses of Lazarus's theory and the empirical literature in the area, our results confirmed the contribution of Big Five personality dimensions as a significant set of predictors in accounting for each of the cognitive appraisals examined (e.g., Shewchuk et al., 1999; Vollrath, 2001; Penley and Tomaka, 2002; Kulenović and Buško, 2006; Kaiseler et al., 2012; Schneider et al., 2012). Specifically, emotional stability, conscientiousness, and extraversion showed to have significant independent contributions in explaining the variance of cognitive appraisals. Furthermore, the predictive power of personality was higher with threat and loss appraisals compared with challenge appraisals, which can also be said to be a well-documented finding (e.g., Gallagher, 1990; Shewchuk et al., 1999; Penley and Tomaka, 2002; Schneider, 2004; Tong et al., 2006; Kaiseler et al., 2012; Schneider et al., 2012; Kilby et al., 2018). Emotional stability was shown as a relatively strong independent predictor of loss and, to a lesser extent, threat appraisals, whereas extraversion and conscientiousness proved as significant predictors of challenge appraisal in this research.

The key finding of this study is that the stress mindset contributes to explaining cognitive appraisal variance, above and beyond the contribution of personality. Hence, our results suggest that the stress mindset might have a distinct role in accounting for stress experiences, rather than being merely a manifestation of personality. Thus, our findings add to the recent empirical literature confirming associations between beliefs about stress and perceiving a stressful situation as challenge, threat, and loss (Kilby and Sherman, 2016; Kilby et al., 2018). Moreover, the results showed that personality traits were more important in explaining cognitive appraisals of stress as loss and threat, while the stress mindset has proven to be more important in explaining challenge appraisals. Finally, it is also worth noting that sports competition context variables selected in this study to describe some situational aspects of athletes' potentially stressful experiences did not make any difference in terms of accounting for stress appraisal criteria. Thus, the results generally suggest that stressful experiences in competitive athletes have more in common with their individual dispositions including this newly introduced concept of stress mindset than with situational features describing the intensity of their engagement in sports, such as performance or competition level.

### Study Limitations and Suggestions for Future Research

Several methodological limitations should be mentioned. In the first place, multivariate treatment of the data conducted in answer to the main study questions would preferably call for a larger research sample. Namely, certain inconsistencies and variations in the obtained regression model parameters between the steps of the analyses seem to be at least partly due to the relatively small sample size. Hence, the eventual instability or, in other words, the uncertain size of sampling error attached to the presented regression coefficients could have been reflected in the correctness of the final results and, consequently, in the soundness of the presented interpretations. Moreover, a larger sample would allow for a more accurate estimate of sample heterogeneity, especially in terms of individual and intergroup differences in scores on the scales used in the study depending on the stressor category.

Furthermore, although online questionnaires have certain advantages such as time savings due to ease of distribution and automatic storage of data ready for analysis and minimal costs associated with material distribution (Llieva et al., 2002; Wright, 2005), online questionnaires are accompanied by disadvantages, such as inability to control the participants' focus on completing the questionnaire and the conditions in which participants complete it. It is also difficult to control whether the same participants repeatedly responded to the questionnaire.

In addition, the retrospective nature of this research should also be noted as another possible limitation of this research. Research participants were required to recall their stress experiences over the past 6 months. This time frame may be a possible source of inconsistency. Namely, in the research, we relied on the ability of participants to accurately remember and set in time their feelings and stress experiences. However, it is possible that, for example, some participants referred to a stressor that happened few months before the study and did not remember their stress experience as accurately as participants whose stress problems were closer to the time of participation in the study. Nevertheless, we decided to set a longer time period given the peculiarities of the period in which the research was conducted. Namely, at the time of data collection, trainings and sports competitions were suspended due to COVID-19 crisis for approximately 3 months. However, as mentioned earlier, since the competitive stressors in the research are related to competitive sports in general, i.e., not related to specific competition, even in the absence of competition and training, we deemed that athletes could have struggled with various competitive stressors.

Although this research confirmed the significance of the stress mindset in accounting for cognitive appraisals besides the contribution of general personality dimensions, the extent to which its role is unique in relation to some narrower personality traits known to be associated with stress reactions such as optimism, self-efficacy, and resilience still remains unexplored. Nevertheless, providing that further empirical evidence proves the uniqueness of the stress mindset construct, the findings on its importance for the stress and adjustment processes would be of practical use too. In view of initial experimental evidence (Crum et al., 2013), the insights into the role of stress mindset might thus serve to design effective interventions intended for enhanced coping with stress among athletes.

## Data Availability Statement

The raw data supporting the conclusions of this article will be made available by the authors, without undue reservation.

## Ethics Statement

Ethical review and approval was not required for the study on human participants in accordance with the local legislation and institutional requirements. Written informed consent for participation was not required for this study in accordance with the national legislation and the institutional requirements.

## Author Contributions

DČ contributed to the formulation of the research idea, data acquisition, data analysis and interpretation, and writing of the manuscript. MKV contributed to literature research, data analysis, interpretation of results, and writing of the manuscript. VB contributed to the conceptual design, data analysis, interpretation of results, and writing of the manuscript. All authors contributed to the article and approved the submitted version.

## Funding

This work was partially supported by a grant from the University of Zagreb to VB (Grant No. 11-923-1051) and a grant from the Faculty of Kinesiology to MKV.

## Conflict of Interest

DČ was employed by company Mental Training Center d.o.o. The remaining authors declare that the research was conducted in the absence of any commercial or financial relationships that could be construed as a potential conflict of interest.

## Publisher's Note

All claims expressed in this article are solely those of the authors and do not necessarily represent those of their affiliated organizations, or those of the publisher, the editors and the reviewers. Any product that may be evaluated in this article, or claim that may be made by its manufacturer, is not guaranteed or endorsed by the publisher.
